# Equine Sarcoid: From BPV-Driven Oncogenesis to Host-Sustained Tumor Persistence

**DOI:** 10.3390/pathogens15080847

**Published:** 2026-08-14

**Authors:** Filippo Dell’Anno, Chiara Trebino, Floriana Fruscione, Chiara Grazia De Ciucis, Livia De Paolis, Katia Cappelli, Elisabetta Razzuoli

**Affiliations:** 1 National Reference Center of Veterinary and Comparative Oncology (CEROVEC), Istituto Zooprofilattico Sperimentale del Piemonte Liguria e Valle D’Aosta, 16129 Genova, Italy; floriana.fruscione@izsplv.it (F.F.); chiaragrazia.deciucis@izsplv.it (C.G.D.C.); livia.depaolis@izsplv.it (L.D.P.); 2 Department of Veterinary Medicine, University of Perugia, Via S. Costanzo 4, 06126 Perugia, Italy; katia.cappelli@unipg.it

**Keywords:** equine sarcoid, bovine papillomavirus (BPV), tumor microenvironment, extracellular matrix remodeling, epigenetic reprogramming, host–pathogen interactions

## Abstract

Equine sarcoid is the most common cutaneous neoplasm of equids and represents a distinctive model of virus-associated tumor persistence. Although bovine papillomaviruses, particularly BPV-1 and BPV-2, are recognized as the main etiological agents, viral infection alone does not fully explain the clinical heterogeneity, frequent recurrence, and limited spontaneous regression of these lesions. This review summarizes current evidence on the molecular and cellular mechanisms underlying equine sarcoid pathogenesis, with emphasis on the interaction between BPV infection, host signaling pathways, tumor microenvironment dynamics, and multi-omic evidence of host regulatory networks. BPV oncoproteins, especially E5, promote fibroblast transformation through PDGFβR activation, downstream PI3K/AKT, MAPK and p38 signaling, altered cell survival, and immune evasion mediated by impaired antigen presentation. However, sarcoid persistence appears to depend on broader host-driven processes, including extracellular matrix remodeling, activated fibroblastic and myofibroblastic phenotypes, chronic inflammatory signaling, and ineffective immune clearance. Recent transcriptomic and epigenomic studies further indicate that long non-coding RNAs, DNA methylation changes, circulating microRNAs, and recently identified virus–host chimeric transcripts may contribute to stabilization of the neoplastic phenotype and may represent future biomarkers. Overall, this review proposes a virus-initiated, host-sustained conceptual framework for equine sarcoid pathogenesis, in which viral oncogene activity and host tissue reprogramming cooperate to promote lesion persistence, recurrence, and therapeutic resistance.

## 1. Introduction

Equine sarcoid is the most common cutaneous neoplasm of equids and is described as a fibroblastic, locally invasive, non-metastatic lesion, characterized by high persistence and frequent recurrence after treatment [1,2,3,4,5,6]. It can develop in horses, donkeys, mules and zebras [7,8], has a worldwide distribution, and has a relevant impact on animal welfare, especially when lesions involve functionally delicate sites such as the limbs, eyelids, perioral regions, ventral abdomen, prepuce or areas exposed to friction [4,9,10,11]. Horses of all breeds and colors can develop disease at any age, mostly presenting a first lesion between 2 and 9 years of age. There is no demonstrated sex predilection, although geldings seem to be overrepresented [12]. From a clinical perspective, sarcoids are traditionally classified according to the Knottenbelt classification into six main forms: occult, verrucous, nodular, fibroblastic, mixed and malevolent [11,13]. The different clinical forms may show variable biological behavior: some lesions remain stable for long periods, whereas others may progress toward more aggressive forms, especially following trauma, incomplete surgical manipulation or ineffective treatments [14,15]. This clinical heterogeneity represents one of the most relevant aspects of the disease, as it influences prognosis, therapeutic choice, and recurrence risk.

Sarcoid development is considered multifactorial. In addition to the presence of the BPV virus, several predisposing factors may contribute to lesion development and progression. Cutaneous trauma is regarded as a possible triggering event, as it may allow viral access to subepidermal fibroblasts and initiate abnormal proliferative processes or altered tissue repair [16,17,18,19]. Insect bites have also been proposed as possible mechanical or vectorial factors, capable of favoring viral transmission or inoculation through cutaneous microlesions [1,4,12,17]. Furthermore, although the mechanisms of viral transmission are not yet fully understood, indirect spread through contaminated fomites, tack and grooming equipment has also been proposed as a potential route of infection [20]. Evidence has also raised the possibility of vertical BPV transmission in equids. Support for this possibility was provided by a study reporting the detection of BPV-1 in a congenital papilloma from a newborn foal. In this lesion, a high viral DNA load together with transcription of viral oncogenes and the L1 gene was demonstrated, indicating biologically active infection [21]. Moreover, BPV DNA has been detected in placental tissue from sarcoid-free mares and in blood samples from their respective foals collected before colostrum intake, suggesting that prenatal transmission may occur, although this hypothesis requires further confirmation [19,22].

Moreover, the host genomic background appears to modulate individual susceptibility to sarcoid development, as suggested by associations with variants within the equine major histocompatibility complex and by recent evidence indicating a combined contribution of genes involved in adaptive and innate immune responses [23,24].

Despite substantial progress, key aspects of sarcoid biology remain unresolved, including the relationship between viral genotype and clinical phenotype, the determinants of immune failure despite detectable anti-viral responses, and the lack of reliable biomarkers for prognosis and therapeutic stratification. In particular, the disconnect between persistent immune recognition and failure of tumor clearance remains one of the most intriguing and poorly understood features of the disease.

In this context, the present review synthesizes current knowledge on the molecular and cellular mechanisms underlying equine sarcoid, focusing on the interplay between BPV infection, host signaling pathways, tumor microenvironment dynamics, and multi-omic regulatory layers. By integrating virological, immunological, and systems biology perspectives, we propose a conceptual framework in which equine sarcoid emerges as a virus-initiated but host-sustained fibroblastic disorder driven by dysregulated tissue repair, immune modulation, and epigenetic reprogramming.

This manuscript was conducted as a narrative review aimed at critically summarizing the current evidence on the molecular pathogenesis of equine sarcoids rather than as a formal systematic review. Literature searches were performed using PubMed, Scopus, Web of Science, and Google Scholar. The final literature search was conducted on June 2026.

Representative search terms included combinations of equine sarcoid, bovine papillomavirus, BPV-1, BPV-2, *Deltapapillomavirus*, *E5*, *PDGFβR*, immune evasion, tumor microenvironment, extracellular matrix, transcriptomics, epigenetics, microRNA, long non-coding RNA, DNA methylation, fibroblast transformation, and multi-omics.

Original research articles, reviews and relevant experimental studies published in English were considered. Priority was given to studies published between 2021 and 2026, reflecting the rapid expansion of transcriptomic, epigenetic and systems biology research in this field. However, landmark studies published before 2021 were deliberately retained whenever they provided the original evidence supporting fundamental concepts such as BPV etiology, viral oncogene function, immune evasion mechanisms, clinical classification, or experimental models.

Studies were screened based on their relevance to the molecular mechanisms underlying equine sarcoid pathogenesis. Reports focused exclusively on clinical case descriptions, therapeutic outcomes without mechanistic insights, or lacking molecular or pathogenetic data were generally excluded unless they provided essential background information.

## 2. Viral Etiology and BPV Variability

### 2.1. BPV Types Associated with Equine Sarcoids

The viral etiology of equine sarcoid is mainly associated with BPVs belonging to the genus *Deltapapillomavirus*. BPV-1 and BPV-2 are considered the main viral types involved in disease pathogenesis [25], whereas BPV-13 has increasingly been reported in some cases and is now considered among the possible genotypes associated with sarcoids [9,25,26,27,28,29]. More recently, BPV-14 has also been included in molecular investigations of sarcoids, although its pathogenetic role remains to be clarified [30,31] (Table 1).

A distinctive feature of bovine *Deltapapillomavirus* is its ability not to be strictly species-specific. Indeed, whereas in cattle BPV-1 and BPV-2 mainly induce cutaneous fibropapillomas that are generally benign and often regress, in equids they are associated with persistent, locally invasive fibroblastic lesions with limited tendency to spontaneous regression [32,33]. The most recent genomic analyses support the hypothesis that interspecies transmission between cattle and equids is an ongoing process, rather than the result of an ancient introduction followed by the circulation of variants stably adapted to the horse [32].

Molecular investigations have also highlighted greater virological complexity than the traditional model based exclusively on BPV-1 and BPV-2. Recent studies based on droplet digital PCR have confirmed the predominance of BPV-1 in several case series, but have also documented frequent co-infections, especially BPV-1/BPV-2, and the less common presence of BPV-13 and BPV-14 [27]. However, genotype distribution appears to vary according to geographical area: for example, a screening conducted on a European population of sarcoid-bearing equids did not detect BPV-13 in the analyzed samples, suggesting a low occurrence of this genotype in specific geographical contexts [34]. Similarly, studies conducted in Japan and Egypt have indicated that BPV genetic variability may reflect geographical origin more than host species, reinforcing the idea of shared viral circulation between cattle and equids [9,25].

Despite these advances, the correlation between viral genotype, viral load, co-infections and clinical phenotype of the lesion remains poorly understood. Some studies have shown that viral load and early viral gene expression may vary among clinical subtypes, but they do not appear to fully explain the different aggressiveness or biological behavior of the lesions [32,35,36,37]. This suggests that the clinical variability of sarcoids depends not only on the virus, but also on its interaction with host factors, the tumor microenvironment, the immune response and local molecular regulation.

**Table 1:** BPV genotypes associated with equine sarcoids: distribution and evidence of involvement.

BPV Genotype	Viral DNA in Equine Sarcoids	Active Transcription/Protein Expression	Functional Evidence	Prevalence /Frequency	Role in Pathogenesis	Geographical Distribution	References
BPV-1	Frequently detected by PCR/ddPCR/sequencing	BPV RNA, RT-PCR and E5 expression demonstrated; strongest evidence among BPVs	Experimental inoculation induces sarcoid-like lesions. E5 transforms fibroblasts via PDGFβR. Natural sarcoids are considered non-productive	Prevalence: 1–12% worldwide (35–90% of dermatological neoplasms)	Main oncogenic driver; E5-mediated fibroblast transformation.	Worldwide	[25,27,28,32,38]
BPV-2	Frequently detected alone or with BPV-1; predominant in several North American and New Zealand cohorts	RNA/E5 reported, but evidence weaker than for BPV-1	Experimental evidence supports pathogenicity; no genotype-specific transformation assays	Frequency: Austria 71.0%; Japan 30%; New Zealand 88.3%; BPV1/BPV2 co-infections 9.6%	Similar oncogenic potential to BPV-1; may promote persistence	Worldwide	[25,27,28,32]
BPV-13	Detected by PCR in Brazil, Southern Italy, and low-copy mixed infections detected by ddPCR in Austria	Not demonstrated	No infectivity or transformation studies	Frequency: Brazil 20%; Europe 0%; Austria 5.4% (mixed infections only); Southern Italy 19.5%.	Possible contributory role; unclear oncogenic potency in equids	Sporadic reports	[29,34,39]
BPV-14	Low-copy DNA detected by ddPCR only in mixed infections	Not demonstrated	No functional studies in equine sarcoids	Frequency: Austria 10.8%; Southern Italy 0%.	Uncertain role	Limited reports	[27,30]
Mixed infections	Co-detection varies with region and detection method	No genotype-specific transcription/protein data	No functional studies; association with severe disease remains preliminary	Frequency: Austria 67.7% BPV1/BPV2; New Zealand 9.6% BPV1/BPV2; Brazil: BPV1, BPV2 and BPV13	May contribute to heterogeneity and persistence.	Reported worldwide	[27,29,31]

### 2.2. Cross-Species Transmission and Molecular Mechanisms of Host Adaptation

Papillomaviruses are generally characterized by a remarkable degree of host specificity, reflecting a long evolutionary history of virus–host co-speciation. Consequently, the ability of bovine papillomavirus types 1 and 2 (BPV-1 and BPV-2) to infect equids represents one of the few well-documented exceptions within the *Papillomaviridae* family. Unlike most papillomaviruses, which complete their productive life cycle exclusively in their natural host, BPV establishes persistent infections in equine fibroblasts and gives rise to equine sarcoids, making these viruses a unique model for studying papillomavirus cross-species transmission [1,19].

Although BPV-associated equine sarcoids have been recognized for several decades, the mechanisms enabling bovine papillomaviruses to overcome the species barrier remain incompletely understood. The most comprehensive study investigating the evolutionary origin of BPV infection in horses was performed by Trewby and collaborators [40], who analysed the long control region (LCR) of BPV-1 from 116 equine sarcoids and 15 bovine papillomas collected across four continents. Their phylogenetic analyses demonstrated that BPV-1 isolates from horses do not form a distinct equine-specific lineage but are interspersed among bovine isolates, indicating that equine sarcoids most likely arise from multiple independent bovine-to-equine transmission events rather than from the emergence of a horse-adapted BPV strain. Furthermore, the viral population exhibited a marked phylogeographical structure, with an ancestral lineage mainly represented in Africa, South America and Australia and a more recently diverged European lineage. Molecular clock analyses suggested that this diversification largely predated the domestication of cattle and horses, indicating that BPV genetic diversity is ancient and unlikely to have originated from a recent host-switching event [40].

Interestingly, Trewby et al. also identified sequence variant 20 (SV20) as the predominant BPV-1 variant in horses, accounting for approximately 43% of all equine isolates and representing the only variant detected across all geographical regions examined. Although the limited number of bovine samples prevented definitive conclusions regarding its prevalence in cattle, the widespread occurrence of SV20 in equids suggested that this variant may possess a selective advantage within the equine host. Previous functional analyses by Nasir and collaborators [41](2007) showed that the LCR of SV20, previously classified as variant II, exhibits higher transcriptional activity than the BPV-1 reference LCR, particularly in equine fibroblasts compared with bovine fibroblasts. This finding supports the hypothesis that variation within viral regulatory regions may influence BPV-1 fitness in the equine host and contribute to viral persistence after cross-species transmission [40,41].

These findings indicate that adaptation to the equine host is unlikely to depend on the emergence of a single equine-specific viral lineage. Rather, viral adaptation probably reflects the selection of naturally occurring variants that exhibit increased biological fitness in equine cells.

Besides sequence variation within the regulatory LCR, attention has also focused on the viral oncogene E5, which encodes the major transforming protein of BPV. The first evidence of genetic variability within E5 was provided by Chambers and collaborators [42], who identified naturally occurring E5 sequence variants in equine sarcoids and proposed that amino acid substitutions could modify the biological behaviour of the oncoprotein [42]. Subsequently, Savini and collaborators [43] extended these observations by analysing the E5 gene in BPV-1 isolated from both sarcoid-bearing and subclinically infected horses. While sarcoid samples generally contained a single predominant E5 sequence, clinically healthy horses carried multiple nucleotide variants simultaneously, demonstrating the coexistence of genetically distinct viral populations within individual animals [43].

This genetic heterogeneity revealed substantial intra-host BPV-1 diversity within individual horses. In the original study, the authors proposed that these genetically related viral populations might represent viral quasispecies, a concept classically defined as a dynamic distribution of closely related viral genomes that evolve through continuous mutation and selection within a host. Although viral quasispecies are well established in RNA viruses and have occasionally been reported in DNA viruses, the detection of multiple BPV-1 sequence variants alone does not conclusively demonstrate true quasispecies dynamics. Rather, the coexistence of mutated and non-mutated viral genomes within the same individual primarily supports the presence of considerable intra-host viral genetic diversity. Notably, Savini et al. identified seven different point mutations within the E5 gene, including both synonymous and non-synonymous substitutions, some of which had not been previously described. The coexistence of these variants may provide the substrate upon which natural selection could act during viral adaptation to the equine host.

An intriguing observation made by Savini et al. was that the E5 variants identified in subclinically infected horses differed from those consistently detected in sarcoids. Based on these findings, the authors hypothesized that specific sequence variants may confer a selective advantage in equids and suggested that increasing intra-host viral diversity could represent an intermediate evolutionary stage preceding sarcoid development. Furthermore, they proposed that the coexistence of multiple E5 variants may support the gradual selection of BPV-1 variants better adapted to the equine host, rather than the emergence of a completely distinct equine viral lineage [41]. Whether these genetically diverse viral populations fulfil the criteria of true quasispecies remains to be determined through longitudinal sequencing studies and population genetic analyses.

Taken together, evidence here reported suggests that BPV adaptation to equids is a multifactorial process involving viral genetic diversity, regulatory sequence variation and intra-host evolution of early oncogenes such as E5. Nevertheless, none of these mechanisms has yet been experimentally demonstrated to directly mediate cross-species transmission. Therefore, the molecular determinants enabling BPV to overcome the species barrier remain one of the major unresolved questions in equine sarcoid biology and represent an important area for future investigation.

## 3. Molecular Pathogenesis of Equine Sarcoids

### 3.1. Viral Tropism, Persistence and Oncogenic Drivers

The pathogenesis of equine sarcoid is closely linked to the peculiar tropism of bovine Deltapapillomaviruses (BPV-1 and BPV-2) for dermal fibroblasts, a feature that distinguishes these viruses from most papillomaviruses, which typically display strict epitheliotropism and species specificity [27,33]. In sarcoids, viral DNA is predominantly localized within the fibroblastic component of the lesion, consistent with the mesenchymal nature of the tumor and supporting the concept that fibroblasts represent the principal target cells of transformation [19,44].

Unlike productive papillomaviral infections characterized by complete viral replication and virion release, BPV infection in equine sarcoids is generally considered abortive or poorly productive [19,45,46,47,48,49]. The viral genome persists mainly in episomal form within transformed fibroblasts, contributing to chronic infection and long-term lesion maintenance [50,51,52,53]. Experimental studies further support this model, demonstrating that primary equine fibroblasts infected with BPV-1 or BPV-2 virions acquire sarcoid-like phenotypic features, including hyperproliferation, loss of contact inhibition and sustained viral transcriptional activity [33]. These observations strongly reinforce the hypothesis that persistent fibroblastic infection represents a key initiating event in sarcoid development.

Among BPV early proteins, E5 is considered the major oncogenic driver involved in equine sarcoid formation [46,54,55]. The transforming activity of E5 mainly depends on its interaction with platelet-derived growth factor receptor beta (PDGFβR), whose constitutive activation promotes proliferative and survival signalling in infected fibroblasts [19,54,56]. Interestingly, E5 is the most highly conserved oncoprotein among bovine Deltapapillomaviruses, and its biological activity has been demonstrated in lesions arising in cattle, horses and sheep. This remarkable conservation may partly explain the unique ability of bovine Deltapapillomaviruses to cross species barriers while maintaining activation of conserved host signalling pathways, particularly PDGFβR, although direct evidence linking E5 conservation to host adaptation is still lacking [55,57]. In addition to its oncogenic role, E5 also contributes to immune evasion through interference with MHC-I expression and intracellular trafficking, thereby reducing antigen presentation and favoring immune escape of infected cells [46,54,58]. The identification of antibodies directed against E5 in affected horses further highlights the immunological relevance of this protein and suggests its potential utility as a diagnostic or immunotherapeutic target [46].

The E6 and E7 proteins also appear to contribute to transformation by modulating cell-cycle regulation, adhesion and survival pathways, although their role seems secondary compared with the central oncogenic activity of E5 [19,33,54]. Overall, equine sarcoid pathogenesis emerges as the consequence of persistent viral infection combined with oncogene-mediated deregulation of fibroblast biology, metabolic reprogramming, and incomplete immune clearance [12,59].

### 3.2. Intracellular Signaling Pathways Involved in Sarcoid Transformation

At the molecular level, equine sarcoid development appears to result from the convergence of multiple interconnected signaling pathways activated or deregulated by BPV oncogenes. Recent evidence also suggests the involvement of metabolic reprogramming mechanisms in sarcoid biology. Overexpression of Carnitine Palmitoyl Transferase 1A (CPT1A), a key regulator of fatty acid β-oxidation, indicates that altered energy metabolism may contribute to the maintenance and survival of transformed fibroblasts [59]. Among these, the PDGFβR axis represents one of the central molecular hubs. Interaction between E5 and PDGFβR induces receptor phosphorylation and constitutive activation, promoting pathological proliferation, morphological alterations and enhanced motility of transformed fibroblasts [19,54,55,60] (Table 2).

Activation of PDGFβR is associated with stimulation of downstream signaling cascades involved in proliferation, apoptosis resistance and cellular transformation. In particular, the PI3K/AKT pathway contributes to regulation of survival, anoikis resistance and cell-cycle progression, whereas activation of Ras-MAPK-ERK signaling has been associated with enhanced proliferative and migratory behavior in sarcoid-derived cells. In parallel, the JNK/AP-1 axis may contribute to transcriptional activation of genes associated with invasion and extracellular matrix remodeling. Furthermore, both E5 and E6 have been linked to activation of p38 MAPK signaling, which appears important for fibroblast transformation and maintenance of the neoplastic phenotype [19,54,60] (Table 2).

The biological effects of these signaling pathways extend beyond proliferation alone and involve profound alterations in cellular homeostasis and survival. Dysregulation of p53 has been proposed as a possible contributor to sarcoid progression, although available evidence remains heterogeneous. Rather than being associated with recurrent TP53 mutations, p53 dysfunction in sarcoids may depend on altered intracellular localization and impaired transcriptional activity [19,28,61,62]. In addition, selective expression of BAG3 in sarcoid tissues and BPV-positive cell lines suggests the activation of anti-apoptotic mechanisms that may further enhance survival of transformed fibroblasts [19,63].

Recent evidence also suggests the involvement of altered autophagy-related pathways in sarcoid biology. Increased expression of Beclin-1, LC3 and p62 indicates dysregulation of autophagy-associated markers, which may contribute to the survival of transformed fibroblasts and their adaptation to the tumor microenvironment. However, since these findings are based on static marker expression, further functional studies assessing autophagic flux are required to clarify whether autophagy is truly impaired during equine sarcoid development [64].

Additional molecular alterations involve angiogenic and epigenetic pathways. Increased HIF-1α expression has been associated with hypoxia-related signaling and angiogenesis, suggesting that oxygen homeostasis and neovascularization may contribute to sarcoid progression [19,65,66]. Epigenetic abnormalities have also been described, including hypermethylation of genes involved in tumor suppression and cell-cycle regulation, such as S100A14, SFN/stratifin and SPARC. These observations support the hypothesis that DNA methylation contributes to the molecular reprogramming of neoplastic fibroblasts. Nevertheless, the currently available evidence remains largely correlative, and functional studies are required to determine whether these epigenetic alterations actively maintain the transformed phenotype or reflect secondary consequences of BPV-driven transformation [19,67].

Although less extensively investigated, additional regulatory pathways may also participate in sarcoid biology. In particular, Wnt/β-catenin and Hippo/YAP/TAZ signaling represent biologically plausible candidates because of their established role in mechanotransduction, proliferation and cell–matrix interactions in other tumor models. However, current evidence in equine sarcoids remains limited, and these pathways should presently be considered emerging areas for future investigation rather than fully established pathogenetic mechanisms [68] (Table 2).

**Table 2:** Molecular pathways involved in equine sarcoid pathogenesis.

Pathway/Mechanism	Main Viral or Cellular Mediator(s)	Biological Effects in Sarcoids	Evidence/Relevance	References
Metabolic reprogramming/fatty acid β-oxidation	CPT1A overexpression	Enhanced lipid utilization, metabolic adaptation and survival of transformed fibroblasts	Emerging evidence suggesting metabolic involvement in sarcoid biology	[59]
PDGFβR activation	BPV E5	Constitutive receptor phosphorylation, fibroblast proliferation, increased motility and transformation	Central oncogenic axis in sarcoid development	[19,54,55]
PI3K/AKT signaling	PDGFβR downstream activation	Survival signaling, apoptosis resistance, anoikis inhibition, cell-cycle progression	Sustains transformed fibroblast phenotype	[19,69]
Ras-MAPK-ERK pathway	PDGFβR downstream signaling	Cell proliferation and migratory behavior	Activated in sarcoid-derived cell lines	[19,60]
JNK/AP-1 signaling	E5-associated signaling	Transcriptional activation of genes involved in invasion and ECM remodeling	Links oncogenic signaling with stromal remodeling	[19]
p38 MAPK pathway	E5/E6	Fibroblast transformation and proliferation	Contributes to maintenance of neoplastic phenotype	[19,54]
Matrix metalloproteinases (MMPs)	AP-1, VEGF and stromal signaling	ECM degradation, tissue remodeling and invasiveness	Increased MMP-1, MMP-2, MMP-3, MMP-9 and MMP-14 reported	[16,19]
VEGF signaling	Tumor-associated angiogenic signaling	Angiogenesis and possible stimulation of MMP production	Supports invasive stromal phenotype	[19,66]
HIF-1α activation	Hypoxia-related signaling	Angiogenesis and adaptation to hypoxic microenvironment	Associated with tumor progression	[19,65]
p53 dysregulation	Altered localization and transcriptional impairment	Imbalance between proliferation and apoptosis	Functional impairment rather than recurrent mutations	[19,61,62]
BAG3 anti-apoptotic pathway	BAG3 overexpression	Increased cell survival and apoptosis inhibition	Detected in sarcoid tissues and BPV-positive cell lines	[19,63]
Autophagy dysregulation	Beclin-1, LC3, P62	Enhanced fibroblast survival, adaptation to hypoxia and altered collagen production	Impaired autophagic flux reported in sarcoids	[64]
Epigenetic deregulation	DNA methylation changes	Stable alteration of tumor suppressor and ECM-associated genes	Involves S100A14, SFN/stratifin and SPARC	[19,67,70]
Immune evasion	BPV E5	Reduced MHC-I expression and impaired antigen presentation	Promotes persistence of infected cells	[46,54,58]
Emerging pathways	Wnt/β-catenin; Hippo/YAP/TAZ	Potential regulation of mechanotransduction and proliferation	Still poorly characterized in sarcoids	[19]

## 4. Tumor Microenvironment, Immune Modulation and Host Regulatory Reprogramming

### 4.1. Stromal Remodeling and Extracellular Matrix Dynamics

In equine sarcoids, the tumor microenvironment cannot be regarded as a merely passive scaffold supporting neoplastic proliferation. Instead, increasing evidence indicates that extracellular matrix (ECM) remodeling and stromal reorganization are central components of lesion biology. The ECM not only provides structural support, but also regulates migration, adhesion, proliferation, differentiation and survival through continuous bidirectional interactions with resident cells. Within this framework, sarcoids may also be interpreted as the consequence of an aberrant wound-healing process occurring in the presence of persistent BPV infection [44,71,72].

Transcriptomic studies strongly support the importance of stromal remodeling in sarcoid progression. Genes involved in ECM organization and cell adhesion are consistently deregulated both in sarcoid tissues and in fibroblasts experimentally expressing BPV1 E1^E4 constructs, indicating that transformation is associated with profound reprogramming of the stromal niche [16,72]. Alterations in metalloproteinases and ECM turnover further reinforce this concept, suggesting that sarcoid progression depends on active remodeling of the surrounding tissue microenvironment rather than on autonomous proliferation alone.

The stromal compartment of sarcoids is dominated by activated fibroblasts displaying fibroblastic or myofibroblastic features. Immunohistochemical analyses demonstrated diffuse expression of fibroblast activation protein alpha (FAPα) in neoplastic fibroblasts, supporting the concept that activated stromal cells represent an integral component of the tumor program rather than a secondary reactive phenomenon [44]. Chromogenic in situ hybridization demonstrated that BPV nucleic acids were predominantly localized within neoplastic fibroblasts, with no detectable signal in the surrounding non-neoplastic tissue. This observation suggests that epithelial alterations are likely mediated by paracrine signalling arising from the neoplastic stromal compartment rather than by direct viral infection of keratinocytes [44].

### 4.2. Host Genomic and Epigenomic Reprogramming

Recent omics studies have substantially expanded the understanding of equine sarcoid biology, revealing that BPV-driven transformation is accompanied by extensive host transcriptional and epigenetic reprogramming. RNA-seq analyses consistently demonstrate profound deregulation of genes involved in proliferation, apoptosis, immune signalling, cell adhesion and ECM remodelling, supporting the concept that sarcoid progression depends on coordinated reorganization of host cellular networks rather than on viral oncogene activity alone [54,68,70,72].

Attention has recently focused on long non-coding RNAs (lncRNAs), which add an additional regulatory layer to sarcoid pathogenesis. Transcriptomic analyses identified thousands of differentially expressed lncRNAs in sarcoids compared with healthy skin, revealing extensive non-coding RNA deregulation associated with pathways involved in ECM disassembly, proliferation, apoptosis and cancer-related signalling [70]. Functional analyses further identified lncRNA–gene interaction networks involving ECM-associated genes, including members of collagen and ADAMTS families, suggesting that lncRNAs may actively regulate stromal activities and matrix stability during lesion progression.

Integration of transcriptomic and methylomic datasets additionally revealed widespread epigenetic deregulation affecting lncRNA promoters and gene bodies. Several lncRNAs displayed inverse correlations between methylation status and expression levels, indicating that aberrant DNA methylation may contribute to stabilization of the neoplastic phenotype through persistent dysregulation of non-coding regulatory networks [70]. These findings reinforce the concept that sarcoids are not solely virus-driven lesions, but tumors maintained by stable host epigenetic reprogramming.

In addition to tissue-associated transcriptomic alterations, circulating non-coding RNAs are emerging as potential minimally invasive biomarkers in equine sarcoid. Hamza and collaborators [73] investigated three whole-blood microRNAs, eca-miR-127, eca-miR-379 and eca-miR-432, in sarcoid-bearing equids before and after therapy. Although none of these miRNAs were able to predict treatment outcome before therapy, eca-miR-379 and eca-miR-432 decreased over time in horses with successful treatment but not in those with therapeutic failure, suggesting a possible role in longitudinal monitoring rather than true pre-treatment prognostication [73]. A subsequent study by Beermann et al. (2026) further refined this interpretation, showing that eca-miR-432 had moderate diagnostic potential for equine sarcoid, but no reliable prognostic or theragnostic value, and no significant treatment-associated changes [74]. Overall, these findings suggest that circulating miRNAs may complement clinical evaluation as non-invasive biomarkers, particularly for diagnosis or follow-up, but they cannot yet be considered validated tools for predicting lesion behavior or guiding therapeutic decisions [73,74].

Among the currently available candidates, the BPV E5 oncoprotein probably represents the biomarker with the greatest short-term translational potential because of its dual biological and clinical relevance. Besides acting as the principal viral oncogenic driver, E5 elicits measurable humoral immune responses in affected horses and may therefore represent both a diagnostic biomarker and a potential immunotherapeutic target. By contrast, circulating microRNAs appear promising as minimally invasive biomarkers for disease monitoring, whereas lncRNAs and DNA methylation signatures currently remain valuable research tools requiring further clinical validation in large prospective cohorts.

An additional emerging area concerns virus–host chimeric transcripts. Virus–host chimeric transcripts are hybrid RNA molecules generated by transcriptional fusion between viral and host genomic sequences. Such transcripts have been identified in several virus-associated human cancers and, more recently, have also been detected in equine sarcoids through integrated transcriptomic analyses [68]. Although their biological significance remains to be fully elucidated, these molecules may represent an additional mechanism of BPV–host interaction and could contribute to transcriptional deregulation, viral persistence and tumor progression. Their identification also opens new perspectives for the development of molecular biomarkers and for understanding long-term virus–host interactions in equine sarcoids.

Taken together, current omics data support a multilevel model of sarcoid pathogenesis in which BPV infection acts as the initiating oncogenic event, whereas lesion persistence and progression depend on complex interactions among stromal remodeling, immune modulation, transcriptional deregulation and epigenetic stabilization of the transformed phenotype (Table 3).

**Table 3:** Tumor microenvironment, immune modulation and host reprogramming interactors in equine sarcoids.

Biological Component	Main Findings	Proposed Role in Sarcoid Biology	References
Extracellular matrix (ECM) remodeling	Deregulation of ECM organization and adhesion pathways	Promotes stromal reorganization, invasion and lesion maintenance	[16,71,72]
Altered wound-healing response	Persistent fibroblastic activation in BPV-positive tissue	Contributes to chronicity and abnormal stromal repair	[72]
Activated fibroblasts/myofibroblasts	FAPα-positive neoplastic fibroblasts	Indicates active stromal participation in tumor progression	[44]
Tumor–epidermis interaction	BPV restricted to neoplastic fibroblasts	Suggests paracrine stromal signaling rather than epithelial infection	[44]
Chronic inflammatory microenvironment	Coexistence of IL6, IL1β, IL10, CCL5, TNF and TGFB1 dysregulation	Supports fibroblast activation, immune modulation and tumor persistence	[35]
Macrophage–fibroblast cross-talk	Cytokine-mediated reciprocal activation	May enhance proliferation, inflammation and lesion progression	[35]
Humoral immune response	Antibodies against sarcoid cells and E5 detected in affected horses	Immune recognition occurs but is insufficient for viral clearance	[32,46]
Transcriptomic deregulation	Altered expression of genes related to proliferation, apoptosis, adhesion and immunity	Reflects extensive host-cellular reprogramming	[16,54,70]
lncRNA deregulation	Thousands of differentially expressed lncRNAs identified	Regulatory role in ECM remodeling, proliferation and migration	[70]
lncRNA–ECM interactions	lncRNAs associated with collagen and ADAMTS genes	Potential modulation of stromal remodeling and matrix stability	[70]
Epigenetic reprogramming	Differential methylation of lncRNA-associated regions	Stabilization of the neoplastic phenotype	[70]
Circulating microRNAs	Differential expression of eca-miR-127, eca-miR-379 and eca-miR-432 in whole blood	Potential prognostic and treatment-monitoring biomarkers	[73]
Virus–host chimeric transcripts	Recently identified in equine sarcoids by integrated transcriptomic analysis	Potential contribution to transcriptional deregulation and biomarker discovery	[68]
Multi-omic host reprogramming	Integration of transcriptomic and methylomic alterations	Indicates systems-level remodeling sustaining tumor progression	[68,70,72]

### 4.3. Immune Microenvironment and Immune Evasion

The persistence and recurrence of equine sarcoids are closely linked to the inability of the equine host to mount a fully effective antiviral immune response. Unlike BPV-induced fibropapillomas in cattle, which frequently regress spontaneously, equine sarcoids are typically chronic, recurrent and poorly prone to spontaneous resolution, suggesting the establishment of a permissive immunological microenvironment [12,19,26,32].

Consistent with this view, spontaneous regression of naturally acquired equine sarcoids is considered an exceptional event rather than a common biological outcome [75]. Brandt recently emphasized that, although experimental BPV-induced pseudo-sarcoids may regress following the induction of a robust cellular immune response, naturally occurring sarcoids usually persist, likely because of low viral antigen availability, persistent episomal BPV infection and viral immune evasion mechanisms [75]. Reports of spontaneous regression in natural disease are limited and appear to involve mainly mild occult or verrucous lesions, often with incomplete histopathological or molecular confirmation [75]. These observations further support the hypothesis that sarcoid persistence depends not only on fibroblast transformation, but also on an immune-permissive microenvironment unable to achieve effective BPV clearance and tissue normalization [75].

One of the principal mechanisms of immune evasion is mediated by the E5 oncoprotein, which interferes with MHC-I expression and antigen presentation, thereby reducing recognition of infected cells by cytotoxic T lymphocytes [46,54,58];. Nevertheless, sarcoid-affected horses can generate humoral immune responses against both autologous and allogeneic sarcoid cells, indicating that tumor-associated antigens remain immunologically recognizable despite incomplete viral clearance [51]. Similarly, antibodies directed against E5 confirm the immunogenicity of this viral protein, although their protective significance remains unclear [46].

The inflammatory landscape of equine sarcoids further highlights the heterogeneity of the tumor microenvironment and appears to vary according to lesion phenotype. Rather than representing a uniform inflammatory signature shared by all sarcoids, cytokine dysregulation seems to be partly subtype-dependent. Parkinson et al. (2024) showed that IL6 and IL1β were the only cytokines significantly differing between sarcoid subtypes, with higher expression in fibroblastic lesions compared with occult and nodular sarcoids [35]. This exaggerated pro-inflammatory profile may reflect the aggressive, granulation tissue-like nature of fibroblastic sarcoids and could be influenced by secondary surface contamination or neutrophilic inflammation, which are common in this lesion type [33]. However, alternative sources of IL6 and IL1β, including activated fibroblasts and injured keratinocytes, should also be considered, as both cytokines may contribute to macrophage–fibroblast crosstalk, fibroblast activation, cytokine amplification and recruitment of additional inflammatory cells [35].

Conversely, IL10 and CCL5 were increased across sarcoid subtypes compared with normal skin, suggesting the presence of a broader immunoregulatory component within the sarcoid microenvironment [35]. This interpretation is supported by previous studies describing increased FOXP3 expression in sarcoid-associated tissues and an immunosuppressive cytokine milieu involving both tumor cells and infiltrating T lymphocytes, suggesting that regulatory immune mechanisms may contribute to ineffective viral clearance and lesion persistence [8,76]. In contrast, TNF and TGFB1 showed significant upregulation mainly in occult lesions compared with normal skin, although their expression did not significantly differ between sarcoid subtypes [35]. These findings indicate that inflammation in sarcoids should not be interpreted as a single homogeneous process, but rather as a lesion-type-dependent feature of the tumor microenvironment. Moreover, quantitative immunohistochemical analyses have demonstrated increased CD4+ and CD8+ T-cell infiltration in sarcoid and perilesional tissues, indicating that cytokine dysregulation occurs within a broader remodeling of the local immune-cell compartment [77]. In this context, chronic inflammation and immune regulation may contribute differently to viral persistence, fibroblastic proliferation, immune modulation and lesion progression depending on the clinical phenotype of the sarcoid [35,76,77].

### 4.4. Immune Escape: Therapeutic Implication

Treatment of equine sarcoids remains challenging, and no universally effective therapeutic approach is currently available. Recurrence is frequent, particularly after incomplete excision or traumatic manipulation of the lesion, supporting the concept that sarcoid treatment should not be considered only as local tumor removal, but also as an attempt to counteract the immune evasion implement by tumor cells, a process facilitated by an immune-suppressive tumor microenvironment that sustains lesion maintenance [14,19,54,78,79].

Immunotherapy represents a biologically attractive strategy because equine sarcoids arise from persistent infection by BPV and are characterized by incomplete immune clearance of infected fibroblasts. The observation that sarcoid-bearing horses can develop antibodies against autologous and allogeneic sarcoid cells suggests that at least some tumor-associated antigens are immunologically recognizable, providing a rationale for antigen-directed immunotherapeutic approaches [51]. However, the clinical relevance of this humoral response remains unclear, and the mechanisms determining protective versus ineffective immune recognition are still poorly defined.

The presence of an immunosuppressive TME suggests the use of local therapies capable of modifying the TME by shifting the immune response from cold to hot; Considering the immunosuppressive properties of viral oncoproteins, they could be an interesting mark.

Among potential target proteins, the E5 oncoprotein is particularly relevant because it combines pathogenetic and immunological significance. E5 is considered the major transforming protein of BPV and contributes to both fibroblast transformation and immune evasion, mainly through activation of PDGFβR-dependent pathways and interference with MHC-I antigen presentation [19,46,50,52,53]. The detection of antibodies against E5 in sarcoid-affected horses further supports the immunogenicity of this viral protein and suggests its potential as a diagnostic or immunotherapeutic target [46]. Nevertheless, current evidence is still insufficient to translate E5 targeting into a validated vaccine strategy, and further studies are needed to determine whether E5-directed immunity can induce effective tumor control.

Within this framework, Bacillus Calmette–Guérin (BCG) immunotherapy holds an important place as one of the historically most studied immunotherapeutic approaches for equine sarcoids. BCG immunotherapy can be interpreted as an attempt to reshape the local immune microenvironment rather than as a BPV-specific antiviral treatment. After intralesional inoculation, BCG is recognized by antigen-presenting cells, particularly macrophages and dendritic cells, through TLR2 and TLR4; this triggers a pro-inflammatory response that favors polarization of macrophages toward the M1 phenotype, with release of TNF-α, IFN-γ, nitric oxide and reactive oxygen species, mediators responsible for direct cytotoxic effects on sarcoid fibroblasts and for the creation of a pro-inflammatory tumor microenvironment [78,80,81]. In parallel, exposure of dendritic cells to BCG increases antigen presentation and promotes activation of CD4+ helper T lymphocytes and, subsequently, CD8+ cytotoxic T lymphocytes; moreover, CD8+ infiltration into the tumor has been correlated with clinical regression of sarcoids. BCG also induces local granulomatous inflammation capable of disrupting the BPV-associated immunosuppressive microenvironment and contributing to restoration of immune surveillance [78,80,81]. This mechanism may counterbalance the immune-suppressive and poorly immunogenic environment of sarcoids by increasing antigen presentation, macrophage activation and recruitment of effector lymphocytes. Therefore, the biological relevance of BCG lies in its ability to convert a chronically persistent lesion into a more immunologically active microenvironment. However, clinical responses remain variable and are influenced by lesion type, anatomical site, treatment protocol and recurrence risk, limiting standardization and broad therapeutic predictability [78].

More recently, molecular approaches targeting viral oncogenes have provided additional proof-of-concept evidence that BPV-dependent mechanisms can be therapeutically exploited. CRISPR/Cas9-mediated editing of BPV-1 episomes in primary equine sarcoid fibroblasts has been shown to successfully target viral regions including E5, E6 and the long control region, resulting in reduced BPV-1 load. Although this approach is not yet clinically applicable, it reinforces the concept that persistent episomal BPV genomes and viral oncogenes represent rational targets for future pathogenetically oriented therapies.

Overall, immunotherapy for equine sarcoids should be viewed as a strategy aimed at overcoming BPV-mediated immune evasion and restoring effective immune control of infected fibroblasts. Current evidence supports the biological plausibility of this approach, particularly through E5-directed targeting and immune microenvironment modulation, but clinical translation remains limited by heterogeneous responses, lack of predictive biomarkers and incomplete understanding of host-specific antiviral immunity in horses [12,26,51,78].

## 5. Conclusions and Future Directions

Equine sarcoid represents a unique model of virus-associated tumor persistence in which BPV infection acts as the initiating event, whereas lesion maintenance depends on a complex interplay between viral oncogene activity and host biological responses. Current evidence indicates that BPV, particularly through the E5 oncoprotein, promotes fibroblast transformation by activating proliferative and survival pathways while simultaneously impairing antigen presentation. However, viral infection alone cannot account for the marked clinical heterogeneity, frequent recurrence and limited spontaneous regression that characterize this disease.

Recent advances have shifted the current paradigm from a predominantly virus-centered model toward a more integrated host–virus perspective. Increasing evidence indicates that extracellular matrix remodelling, chronic immune dysregulation, stromal activation and widespread transcriptional and epigenetic reprogramming are fundamental determinants of sarcoid persistence. In this context, transcriptomic and multi-omic studies have revealed that host regulatory networks—including long non-coding RNAs, DNA methylation changes and other epigenetic mechanisms—likely contribute to stabilizing the transformed phenotype long after the initial viral insult.

Despite these advances, several important knowledge gaps remain. The functional significance of many newly identified molecular pathways has yet to be experimentally validated, and the respective contributions of viral genotype, host genetics and tumor microenvironment to disease progression remain incompletely understood. Although inflammatory mediators have been extensively investigated, the cellular composition of the sarcoid immune microenvironment remains poorly characterized. Comprehensive profiling of immune infiltrates, including T-cell subsets, macrophage polarization, dendritic cells and other stromal immune populations, is still lacking, limiting the understanding of immune escape mechanisms and the identification of novel immunotherapeutic targets.

Likewise, robust biomarkers capable of predicting biological behaviour, therapeutic response or recurrence are still lacking.

The conceptual framework proposed in this review should be regarded as a working biological hypothesis rather than a definitive model of sarcoid pathogenesis. One of the major unresolved questions is whether established sarcoids remain continuously dependent on BPV oncogene activity or progressively become sustained by stable host-derived mechanisms, including stromal remodeling, immune dysregulation and epigenetic reprogramming. Addressing this question will require integrated experimental approaches combining BPV-infected primary equine fibroblasts, advanced three-dimensional organotypic culture systems, longitudinal transcriptomic and epigenomic profiling, and functional manipulation of viral oncogene expression. Such experimental models may ultimately clarify the relative contribution of persistent viral activity and host regulatory networks to tumor maintenance while identifying stage-specific therapeutic targets.

Future research should therefore prioritize integrated multi-omic studies combining virological, transcriptomic, epigenomic, immunological and clinical data in well-characterized cohorts. Such an approach will be essential not only to clarify the mechanisms underlying sarcoid pathogenesis but also to identify clinically relevant biomarkers and novel therapeutic targets.

Ultimately, we propose that viewing equine sarcoid as a virus-initiated, host-sustained conceptual framework provides a more comprehensive perspective for understanding its biology and may facilitate the development of mechanism-based therapeutic strategies targeting both persistent viral oncogene activity and host regulatory pathways.

## Data Availability

No new data were created or analyzed in this study. Data sharing is not applicable to this article.

## Abbreviations

The following abbreviations are used in this manuscript:

BPV	Bovine papillomavirus
BCG	Bacillus Calmette–Guérin immunotherapy
